# Heterogeneity of quality of life in the later stages of first-episode psychosis recovery

**DOI:** 10.1007/s11136-022-03277-x

**Published:** 2022-11-15

**Authors:** E. L. Clarke, K. Allott, J. F. I. Anderson, C. X. Gao, K. M. Filia, E. Killackey, S. M. Cotton

**Affiliations:** 1 Melbourne School of Psychological Sciences, The University of Melbourne, Melbourne, VIC 3010 Australia; 2 Orygen, Parkville, Melbourne, VIC Australia; 3 Centre for Youth Mental Health, University of Melbourne, Parkville, Melbourne, VIC Australia; 4 School of Public Health & Preventive Medicine, Monash University, Melbourne, Australia

**Keywords:** Quality of life, First-episode psychosis, Depression, Social inclusion

## Abstract

**Purpose:**

First-episode psychosis (FEP) is characterised by wide heterogeneity in terms of symptom presentation and illness course. However, the heterogeneity of quality of life (QoL) in FEP is not well understood. We investigated whether subgroups can be identified using participants' responses on four QoL domains (physical health, psychological, social relationships, and environmental) 18-months into the recovery phase of FEP. We then examined the discriminant validity of these subgroups with respect to clinical, cognitive, and functioning features of FEP.

**Method:**

Demographic and clinical characteristics, QoL, cognition, and functioning were assessed in 100 people with FEP at the 18-month follow-up of a randomised controlled trial of Individual Placement Support, which aims to facilitate vocational recovery. QoL was measured using the World Health Organisation’s QoL-BRIEF. A two-stage clustering approach using Ward’s method and Squared Euclidean Distance with a k-means confirmation was conducted. Multinomial logistic regressions were used to establish external validity.

**Results:**

Three QoL subgroups emerged: a ‘good’ subgroup with relatively high QoL across all domains (31%), an ‘intermediate’ subgroup with relatively low psychological QoL (48%) and a ‘poor’ subgroup with markedly low social relationship QoL (21%). Negative symptoms, depressive symptoms, social/occupational functioning, and social inclusion at follow-up predicted subgroup membership. Sensitivity analysis found similar results.

**Conclusion:**

Although some individuals with FEP have QoL comparable to individuals without mental ill health, QoL can remain concerningly low despite treatment efforts. Future research on interventions that target factors associated with poor QoL, such as low social inclusion, is required to counteract prolonged poor QoL in FEP.

**Supplementary Information:**

The online version contains supplementary material available at 10.1007/s11136-022-03277-x.

## Plain English summary

Quality of life has emerged as a valuable outcome in first-episode psychosis (FEP) research. Young people with FEP also consider good quality of life to be more important than symptom management. Until recently, previous research into FEP has sought to identify which clinical, cognitive, and functioning factors are associated with low quality of life in FEP. However, these studies often ignore individual differences in quality of life. In this study we aimed to understand whether young people with FEP, who are in the later stages of their recovery, can be grouped into clinically meaningful subgroups based on their responses on four quality of life domains (i.e., physical health, psychological, social relationships, and environmental). This study indicates that young people with FEP experience either ‘good’, ‘intermediate’, or ‘poor’ quality of life at the later stages of recovery, and that depressive symptoms and social inclusion strongly predict membership to these quality of life subgroups. Findings from this study highlight the possibility of having good quality of life despite the challenges associated with FEP, whilst also demonstrating that poor quality of life can persist despite interventional effort. Future research should focus on developing interventions that address factors associated with poor quality of life, such as low social inclusion.

## Introduction

Compared to individuals without mental ill health, individuals with first-episode psychosis (FEP) report significantly lower levels of quality of life (QoL) [1]. Research has shown that FEP is highly heterogeneous in symptom presentation, severity of clinical course, and functional impact [2]. However, there has been limited investigation into the heterogeneity of QoL in FEP. Ignoring individual differences, prior research commonly adopts the notion of globally diminished QoL in FEP cohorts, focusing on identifying which clinical (e.g. symptoms), cognitive (e.g. verbal fluency), and functioning factors (e.g. employment) are associated with QoL [3].

Understanding variability in QoL in FEP has important clinical implications. For instance, the disparity in QoL between individuals with FEP and the general population persists beyond symptomatic remission, suggesting that psychotic symptoms alone do not produce poor QoL [1]. Moreover, the onset of FEP typically occurs in early adulthood and it has been found that young people with FEP value good QoL as an outcome more highly than symptom management [4]. Consequently, understanding heterogeneity in QoL and its associated features may help shift therapeutic focus from controlling symptoms to improving an individual’s living environment and relationships – all of which are essential facets of QoL.

Liao et al. [5] captured heterogeneity in QoL via cluster analysis to form subgroups of individuals with FEP based on their responses to the World Health Organisation’s [6] QoL Scale-BREF (WHOQoL-BREF) with domains of ‘physical health’, ‘psychological’, ‘social relationships’, and ‘environmental’ QoL. Liao and colleagues [5] found that responses resulted in the clustering of participants into three distinct QoL subgroups (i.e. ‘good’, ‘intermediate’, and ‘poor’), demonstrating that although individuals with FEP can exhibit moderate to concerningly low levels of QoL, some indicate higher levels of QoL, similar to those without mental ill health. Importantly, they found that the subgroups differed significantly from each other on a range of clinical (e.g. depression), cognitive (e.g. semantic verbal fluency), and functional variables (e.g. social inclusion and social/occupational functioning), suggesting that the subgroups are both quantitively and qualitatively unique from each other [5].

However, the presence of QoL heterogeneity at a later stage of FEP recovery remains unclear; Liao et al.’s [5] analysis was limited to participant QoL in the early stages of treatment. Whilst the concept of recovery is contentious, we refer to recovery as one of the three phases of FEP, these include prodromal, acute, and recovery. The recovery phase typically begins a few months after an initial episode and is characterised by a reduction in the intensity and frequency of psychotic symptoms—particularly positive symptoms.

Investigating whether heterogeneity in QoL persists into the later stages of the recovery phase is necessary as research suggests that whilst improvements in QoL may occur, it can remain poor or even deteriorate over time [7]. Moreover, factors associated with QoL in FEP may differ at various stages of the illness [8]. For instance, although there was no relationship between negative symptoms and QoL subgroups at baseline, a relationship may emerge at a later stage of the illness as symptom *persistence*, rather than symptom *severity*, is more strongly associated with QoL in FEP [8, 9].

Using the same sample and methodology as Liao et al. [5], the first aim of this study was to investigate whether homogeneous subgroups of individuals with FEP can be delineated using response to the WHOQoL-BREF [6] at an 18-month follow-up (i.e. post-treatment). The second aim was to validate the QoL subgroups at follow-up by comparing them on a range of clinical, cognitive, and functioning characteristics of FEP. It was hypothesised that three subgroups with distinct QoL profiles would be identified and that the subgroups would differ on clinical, cognitive, and functioning variables.

## Materials and methods

### Design

The current study involved a secondary analysis of baseline and 18-month follow-up data of a single-blinded RCT (Australian and Clinical Trials Registry ACTRN12608000094370), which aimed to investigate the efficacy of Individual Placement and Supporting (IPS) in assisting young people with FEP to achieve vocational recovery [10, 11]. Participants (*n* = 146) were randomised into either 6-months of IPS in addition to treatment-as-usual (TAU) or TAU alone [10, 11]. Assessments were conducted at baseline, 6-months, 12-months, and 18-months. Although the IPS group showed higher rates of employment at the end of treatment, this difference was not seen at the 12- and 18-month follow-up points [11]. The Melbourne Health Human Research Ethics Committee (HREC 2007.648) granted ethical approval for the original study [10, 11] and this secondary analysis.

### Participants

Young people with FEP who had expressed an interest in vocational recovery were approached to participate in the original RCT. Participant recruitment took place at the Early Psychosis Prevention and Intervention Centre (EPPIC), a clinic within the Orygen Specialist Programme in Melbourne, Australia, providing time-limited care. Participants were aged between 15 and 25 years and had experienced a FEP as defined by the Diagnostic and Statistical Manual of Mental Disorders, Fourth Edition-Text Revision [12]. Further inclusion criterion ensured participants had at least 6-months left in the EPPIC programme and had expressed a desire to reach an employment or educational goal. Exclusion criteria included having a severe intellectual disability or experiencing florid psychosis at the time of consent, reducing capacity to provide informed consent, and a lack of English fluency. All participants provided written informed consent and parental/guardian consent in the case of minors under the age of 18 years.

## Measures

### QoL

QoL was measured using the WHOQoL-BREF [6]. This 26-item self-report scale assesses an individual’s perception of their QoL in four domains: physical health (e.g. mobility), psychological (e.g. self-esteem), social relationships (e.g. social support), and environmental (e.g. financial resources). Individual items are measured on a Likert scale ranging from 1 to 5. An overall score for each QoL domain is generated by transforming the sum of the items on a scale from 0 to 100, with higher scores indicative of better QoL. The WHOQoL-BREF has been validated in FEP populations [13] and found to have good internal reliability (α = .85). An overlap between the psychological domain of the WHOQoL-BREF and overall score on the Centre for Epidemiological Studies – Depression Scale (CES-D) was found (Appendix 1).

### Demographics

The demographic characteristics assessed at baseline covered variables such as age, gender, and use of health services.

### Clinical characteristics

Duration of untreated psychosis (DUP) was defined as the time between onset of psychotic symptoms and registration at EPPIC [10]. Clinical diagnosis was established using the Structured Clinical Interview for DSM-IV-TR Axis I Disorders – Patient Edition [14]. Treatment group allocation was recorded and treated as a binary variable (i.e. treatment-as-usual and IPS). Positive symptoms were measured using the four-item positive symptom subscale of the Brief Psychiatric Rating Scale [15], which has demonstrated acceptable internal reliability (α = .77). Negative symptoms were measured using the Scale for the Assessment of Negative Symptoms [16], which was found to have good internal reliability (α = .85). Depressive symptoms were measured using the CES-D[17], with acceptable internal reliability (α = .75).

### Cognition

Based on their association with QoL in FEP [18, 19], semantic verbal fluency and theory of mind (ToM) were the primary cognitive domains of interest. However, IQ was assessed to control for its relationship with verbal fluency [20]. IQ was measured using the ‘Information’ and ‘Picture Completion’ subtests of the Wechsler Adult Intelligence Scale –Third Edition [21]. Semantic verbal fluency was measured using Animal Fluency [22, 23]. ToM was assessed using The Picture Sequencing Task [24], a non-verbal (cartoon) task of ToM. All cognitive assessments were from baseline only.

### Functioning

Social and occupational functioning was measured at follow-up using the single-item Social and Occupational Functioning Assessment Scale [25], which was found to have acceptable internal reliability (α = .77). Employment status was treated as a binary variable (i.e. employed and unemployed) at baseline and follow-up. Social inclusion was assessed via the Social Inclusion Measure (SIM) [26] at baseline and follow-up. The SIM assesses three domains of social inclusion (i.e. social isolation, social relations, and social acceptance) with higher scores indicative of greater social inclusion. The SIM has acceptable internal reliability (α = .77).

## Statistical analysis

All analyses were completed using RStudio (Version 1.4.1717) and were chosen to mirror the methods of Liao et al. [5]. A table of packages used in the analyses can be found in Appendix 2. For the purpose of comparison, QoL clusters at baseline were delineated first. As Liao et al. [5] used IBM SPSS Statistics Version 26, not R Studio (Version 1.4.1717), our baseline cluster solution was expected to differ slightly. As the number of QoL clusters at baseline was established by Liao et al. [5], k-means iterative partitioning with three clusters was conducted. To delineate the follow-up QoL clusters, a two-phase clustering approach was conducted. First, hierarchical agglomerative cluster analyses using Ward’s minimum variance method [27] and Euclidean Distance was completed. The best number of clusters to retain was decided by inspecting dendrograms, the within-cluster sum of squares scree plot, and the principal component analysis (PCA) plot. K-means clustering was then conducted to validate the solution informed by hierarchical clustering. The mean performance of the clusters on each QoL domain was compared using a series of one-way Analysis of Variance (ANOVA), followed by Tukey multiple comparisons. A profile analysis was conducted using the package profileR (Version 0.3–5) to further confirm whether the identified clusters had different profiles in responding to QoL items (e.g. parallel, equal levels, or flatness). [28]. Parallelism is tested using a Multivariate Analysis of Variance and assesses whether the clusters have similar QoL scores to each other. If the clusters are parallel, a test of equality of levels is conducted to determine whether at least one cluster scored higher, on average, across each QoL domain. Finally, the flatness of each cluster was assessed using Hotelling’s T2 test, which was transformed into an F-statistic. Pairwise comparisons for flatness were conducted to identify which cluster had the most within-cluster QoL variation.

The external validity of the QoL subgroups was evaluated using multinominal logistic regressions. A range of clinical, cognitive, and functional variables both at the baseline and follow-up were included in the models. These variables include baseline and follow-up clinical characteristics (DUP, treatment group, positive symptoms, negative symptoms, and depression), baseline cognition (IQ, ToM, and semantic verbal fluency), and follow-up functioning variables (social/occupational functioning, employment status, and social inclusion). A series of univariate multinomial logistic regression models were first conducted for individual variables. If a variable significantly predicted cluster group membership, it was henceforth included in the final multivariate model. For all models, the relative risk ratio (RRR), 95% confidence intervals of the RRR, and the Wald test were calculated to establish the level of association. The alpha (*α*) level was set at .100 for bivariate models and .05 for the multivariate model to ensure the analysis mirrored that of Liao et al. [5]. Adjustment for multiple comparisons was not performed due to the explorative nature of this study, as this adjustment may result in an increased likelihood of type II errors [29].

### Sensitivity analysis

Due to the high overlap between the psychological domain of the WHOQoL-BREF and depression symptoms (Appendix 1), sensitivity analyses were conducted with the psychological domain of the WHOQoL-BREF excluded from the k-means clustering analysis. Further external validation based on the clustering results not considering variations in the psychological domain was then conducted using multinominal logistic regressions.

## Results

### Sample characteristics

In total, 171 young people were assessed for eligibility for the original RCT. Of these 171, 25 were excluded and 146 were randomised. Of the 25 that were excluded, 23 declined to participate and 2 were too unwell to participate. We excluded a further participant from our analysis due to missing QoL data. A detailed description of participant flow can be found elsewhere [11].

The total sample consisted of 145 participants (*M* = 20.41 years, *SD* = 2.38 years). Congruent with the typical FEP cohort [30], most were male, unemployed, and had a schizophrenia spectrum disorder diagnosis Table 1. Of the 145 participants, the 100 with complete follow-up data at 18-months were included in the clustering analysis. There were no major differences between participants who did and did not complete the follow-up assessment (Appendix 3). Consistent with characteristics of the recovery phase of FEP, the participants presented with low positive symptoms at baseline and follow-up Table 1. Additionally, there was a significant reduction in the cohorts’ depressive symptoms and a significant improvement in each QoL domain from baseline to follow-up (Table 1).

**Table 1:** Participant demographics, clinical characteristics, and QoL

Demographics	Baseline	Follow-up	*t*	*df*	*p*-value
	*N* = 145	*N* = 100			
Mean age (*SD*)	20.41 (2.38)	20.80 (2.40)	–	–	–
Gender (%)					
Male	101 (69.18)	66 (66.00)	–	–	–
Female	44 (30.34)	34 (34.00)	–	–	–
Employment status (%)					
Currently in paid work	24 (16.44)	37 (37.00)	–	–	–
Not currently in paid work	121 (82.88)	63 (63.00)	–	–	–
Diagnosis (%)					
Bipolar disorder	20 (13.87)	15	–	–	–
Brief psychotic disorder	1 (.07)	1	–	–	–
Delusional disorder	8 (5.52)	7	–	–	–
Major depressive disorder with psychotic features	17 (11.72)	12	–	–	–
Schizoaffective	19 (13.10)	11	–	–	–
Schizophrenia	55 (37.93)	40	–		–
Schizophreniform	8 (5.52)	6	–	–	–
Psychotic disorder NOS	17 (11.72)	8	–	–	–
Clinical characteristics, mean (SD)					
Positive symptoms (BPRS)	8.46 (4.30)	7.27 (4.81)	1.56	100	.122
Negative symptoms (SANS)	25.33 (12.30)	24.96 (16.04)	.20	100	.845
Depressive symptoms (CES-D)	19.95 (11.70)	14.75 (10.29)	3.60	99	< .001*
QoL					
Physical health	64.68 (16.13)	70.30 (13.60)	−2.29	99	.024*
Psychological	53.36 (19.57)	61.38 (17.66)	−3.71	99	< .001*
Social relationship	57.36 (21.95)	64.67 (22.88)	−2.80	99	.006*
Environmental	61.96 (15.95)	67.16 (16.05)	−2.09	99	.039*

*BPRS* Brief Psychiatric Rating Scale – Positive Symptoms Subscale, *CES-D* Centre for Epidemiological Studies – Depression scale, *SANS* Scale for the Assessment of Negative Symptoms, *NOS* Not otherwise specified

^*^ *p* < .05

### Cluste analysis

Three baseline clusters were delineated via k-means partitioning method for the purpose of comparison with the follow-up clusters. The baseline three-cluster solution included a ‘good’ (*n* = 42), ‘intermediate’ (*n* = 63), and a ‘poor’ QoL cluster (*n* = 40).

**Fig. 1:**
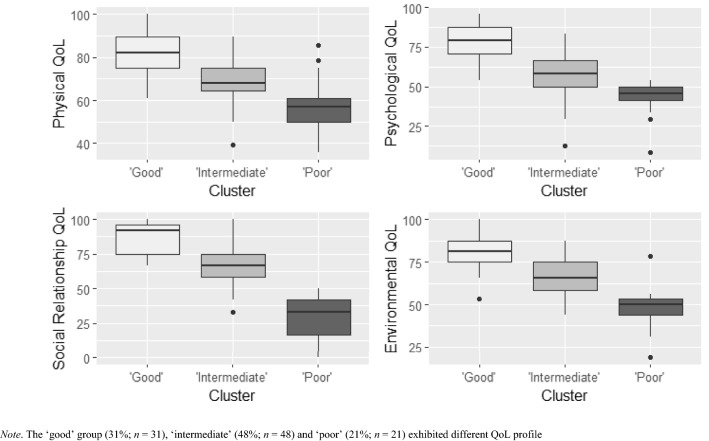
QoL profiles for each cluster group at 18-month follow-up. The ‘good’ group (31%; *n* = 31), ‘intermediate’ (48%; *n* = 48), and ‘poor’ (21%; *n* = 21) exhibited different QoL profile

For the hierarchical agglomerative clustering, an inspection of the dendrogram (Appendix 4) and scree plot (Appendix 5) indicated a three-cluster solution due to its interpretability and good cluster separation. A *k*-means partitioning method also suggested a 3-cluster solution to be optimal and resulted in very similar cluster allocations. The three clusters had distinct QoL characteristics, namely ‘poor’ (*n* = 21), ‘intermediate’ (*n* = 48) and ‘good’ cluster (*n* = 31). Group means for each QoL domain and differences between groups can be observed in Fig. 1.

A series of one-way ANOVAs confirmed that each QoL cluster differed from one another in all four QoL domains (Appendix 6). Post hoc Tukey multiple comparisons can be found in Table 2. A one-way multivariate analysis of variance detected deviations from parallelism, providing strong evidence of an interaction effect between cluster membership and QoL domains, *F*(6, 192) = 5.82, *p* < .001, η^2^ = .15. As parallelism was rejected, a test of equality of levels was not required [27]. The flatness test revealed the presence of significant differences between domains within the groups, *F*(3, 95) = 14.77, *p* < .001, η^2^ = .36. Pairwise comparisons for flatness tests revealed the location of differences variated between subgroups (Table 3).

**Table 2:** Post hoc Tukey multiple comparisons of means for cluster groups against QoL domains

			95% CI		
QoL domain	Cluster contrast	M_*diff*_	LCI	UCI	*p*-adj
Physical health	Intermediate vs. Poor	10.55	3.97	17.14	< .001*
	Good vs. Poor	23.86	16.74	30.97	< .001*
	Good vs. Intermediate	13.30	7.50	19.10	< .001*
Psychological	Intermediate vs. Poor	15.30	7.92	22.69	< .001*
	Good vs. Poor	36.04	28.06	44.02	< .001*
	Good vs. Intermediate	20.73	14.23	27.24	< .001*
Social relationships	Intermediate vs. Poor	31.99	23.78	40.21	< .001*
	Good vs. Poor	52.82	43.94	61.69	< .001*
	Good vs. Intermediate	20.82	13.58	28.06	< .001*
Environmental	Intermediate vs. Poor	17.17	9.98	24.35	< .001*
	Good vs. Poor	31.64	23.88	39.4	< .001*
	Good vs. Intermediate	14.47	8.14	20.80	< .001*

*CI* confidence interval, *LCI* lower confidence interval, *M*_*diff*_ Mean difference, *UCI* upper confidence interval

^*^ = *p* < .001

**Table 3:** Pairwise comparisons for each cluster groups against the QoL domains

				95% CI	
Cluster group	QoL domain	M_*diff*_	*p*-value	LCI	UCI
‘Good’ QoL					
Physical health	Psychological	2.78	.220	−1.76	7.32
	Social relationships	−4.07	.116	−9.20	1.06
	Environmental	0.94	.600	−2.64	4.51
Psychological	Social relationships*	−6.85	.020*	−12.54	−1.17
	Environmental	−1.85	.453	−6.81	3.11
Social relationships	Environmental	5.01	.062	−0.28	10.29
‘Intermediate’ QoL					
Physical health	Psychological*	10.22	< .001*	5.67	14.77
	Social relationships	3.45	.224	−2.19	9.08
	Environmental	2.10	.404	−2.92	7.12
Psychological	Social relationships*	−6.77	.033*	−12.98	−0.56
	Environmental*	−8.12	.002*	−13.02	−3.21
Social relationships	Environmental	−1.35	.613	−6.65	3.96
‘Poor’ QoL					
Physical health	Psychological*	14.97	< .001*	10.43	19.51
	Social relationships*	24.89	< .001*	15.88	33.90
	Environmental*	8.72	.016*	1.81	15.62
Psychological	Social relationships*	9.92	.016*	2.09	17.75
	Environmental	−6.25	.067	−12.99	0.49
Social relationships	Environmental*	−16.17	< .001*	−24.38	−7.96

*CI* confidence interval, *LCI* lower confidence interval, *M*_*diff*_ Mean difference, *UCI* upper confidence interval

^*^ = *p* < .05

As can be seen in Fig. 1 and Table 3, the ‘good’ QoL cluster was characterised by its high QoL and relatively flat profile across all four QoL domains. Contrastingly, the ‘poor’ cluster was characterised by consistently low QoL, with markedly low social relationships QoL. The ‘intermediate’ cluster fell between the ‘good’ and ‘poor’ clusters, whereby participants exhibited average physical health QoL, yet comparatively poor psychological QoL.

A chi-square test of independence was used to compare the cluster sizes at baseline to follow-up. Accordingly, the size of the follow-up clusters did not significantly differ from the cluster sizes at baseline, *x*^*2*^(2) = 1.38, *p* = .501. Furthermore, a chi-square test of independence revealed that cluster membership at baseline was significantly associated with cluster membership at follow-up, *x*^*2*^(4) = 14.12, *p* = .007. A comparison of QoL characteristics of the baseline and follow-up clusters can be found in Appendix 7.

### External validity

The demographic, clinical, cognitive, and functioning characteristics of the three cluster groups are presented in Table 4. External validity was established by comparing the cluster groups using multinomial regression with the ‘good’ group selected as the reference category as this group had relatively high QoL. Bivariate models comparing the clusters on various baseline variables revealed significant group differences in DUP, positive symptoms, and depression at baseline (Appendix 8). However, negative symptoms, treatment group, and cognitive functioning at baseline were not associated with the cluster membership. Bivariate models comparing the clusters on follow-up variables revealed significant group differences among positive symptoms, negative symptoms, depressive symptoms, social and occupational functioning, and social inclusion at follow-up (Appendix 9). However, employment status at follow-up was not associated with cluster membership.

**Table 4:** Key sample characteristics of the follow-up QoL cluster groups

Measure	‘Good’ (*n* = 31)	‘Intermediate’ (*n* = 48)	‘Poor’ (*n* = 21)
Baseline demographics			
Age, mean (*SD*)	20.35 (2.48)	21.12 (2.28)	20.78 (2.41)
Gender, male (%)	20 (64.52)	33 (68.75)	13 (61.90)
Baseline clinical characteristics			
*DUP,* mean	144.00 (199.70)	265.40 (438.56)	365.05 (511.08)
Treatment group, vocational (%)	15 (48.39)	26 (54.17)	13 (61.90)
Follow-up clinical characteristics			
Positive symptoms (BPRS), mean (*SD*)	5.84 (3.77)	6.46 (3.50)	10.48 (6.15)
Negative symptoms (SANS), mean (*SD*)	18.00 (12.76)	24.71 (15.52)	35.43 (16.79)
Depressive symptoms (CES-D), mean (*SD*)	7.03 (4.54)	14.90 (8.94)	25.82 (9.19)
Follow-up functioning			
Currently employed (%)	13 (41.94)	18 (37.50)	6 (28.57)
Social/occupational functioning (SOFAS), mean (*SD*)	63.23 (14.68)	58.44 (14.14)	52.29 (12.38)
Social inclusion, mean (*SD*)	46.61 (5.40)	40.38 (6.27)	34.86 (6.37)
Baseline cognition			
IQ, mean (*SD*)	94.39 (17.36)	93.52 (14.79)	91.81 (12.89)
ToM, mean (*SD*)	4.60 (1.39)	4.53 (1.18)	4.46 (1.44)
Semantic verbal fluency, mean (*SD*)	19.06 (6.60)	18.33(6.31)	19.06 (5.82)

*BPRS* Brief Psychiatric Rating Scale – Positive Symptoms Subscale, *CES-D* Centre for Epidemiological Studies –Depression scale, *DUP* duration of untreated psychosis, *SANS* Scale for the Assessment of Negative Symptoms, *SOFAS* Social and Occupational Functioning Assessment Scale, *ToM* Theory of Mind. Measures that are in italics are premorbid variables, taken at baseline

A multivariate model based on the significant predictors identified in the bivariate models was constructed. The multivariate model indicated that at follow-up negative symptoms, depressive symptoms, social and occupational, and social inclusion independently and significantly predicted membership to the QoL clusters (Table 5).

**Table 5:** Multivariate associations between external variables and follow-up cluster group membership

	Contrast – ‘poor’ vs ‘good					Contrast – ‘intermediate’ vs ‘good’				
		95% CI of OR					95% CI of OR			
Variable	OR	LCI	UCI	Wald	*p*-value	OR	LCI	UCI	Wald	*p*-value
Clinical characteristics										
*DUP*	1.00	1.00	1.00	−.20	.842	1.00	1.00	1.00	−.08	.934
Baseline positive symptoms (BPRS)	1.05	.79	1.38	.34	.734	1.07	.86	1.33	.60	.549
Positive symptoms (BPRS)	1.00	.77	1.27	−.07	.942	.92	.75	1.13	−.79	.432
Negative symptoms (SANS)*	1.11	1.03	1.20	2.62	.009*	1.04	.98	1.09	1.32	.186
Baseline depressive symptoms (CES-D)	1.09	1.00	1.20	1.72	.085	1.02	.96	1.09	.72	.474
Follow-up depressive symptoms (CES-D)*	1.38	1.18	1.61	4.04	< .001*	1.24	1.09	1.41	3.26	.001*
Functioning										
Social/occupational functioning (SOFAS)*	1.12	1.03	1.22	2.55	.011*	1.03	.97	1.08	0.95	.344
Social inclusion*	.74	.64	.86	−3.85	< .001*	.82	.74	.92	−3.45	< .001*

*BPRS* Brief Psychiatric Rating Scale – Positive Symptoms Subscale, *CES-D* Centre for Epidemiological Studies –Depression scale, *CI* confidence interval, *LCI* lower confidence interval, *OR* odds ratio, *SANS* Scale for the Assessment of Negative Symptoms, *SOFAS* Social and Occupational Functioning Assessment Scale, *UCI* upper confidence interval. Measures that are in italics were taken at baseline

^*^ = *p* < .05

### Sensitivity analysis

The sensitivity analysis excluding the psychological domain showed very similar results. A k-means partitioning method also suggested a 3-cluster solution to be optimal and three clusters had distinct QoL characteristics (Appendix 10), namely a ‘poor’ cluster (*n* = 26), ‘intermediate’ cluster (*n* = 51), and ‘good’ cluster (*n* = 23). A series of one-way ANOVAs confirmed that each QoL cluster differed from one another in all three QoL domains included in the clustering algorithm (Appendix 11), and deviations from parallelism (*p* < .001) and flatness (*p* = .005) were also found. The bivariate models comparing the clusters on various baseline and follow-up variables revealed significant group differences in positive symptoms, negative symptoms, depression, social and occupational functioning, and social inclusion (Appendix 12). The multivariate model indicated that at follow-up, depressive symptoms and social inclusion independently and significantly predicted membership to the QoL clusters (Appendix 13).

## Discussion

The current study aimed to extend the findings of Liao et al. [5] by examining whether homogeneous subgroups of QoL can be delineated and validated at a *later stage* of FEP recovery. Three QoL profiles were identified: a ‘good’ subgroup with relatively high QoL in all four domains, an ‘intermediate’ subgroup with a particular deficit in psychological QoL, and a ‘poor’ subgroup with concerningly diminished social relationships QoL. Moreover, depressive symptomology and social inclusion were the strongest predictors of subgroup membership.

## The heterogeneity of QoL in FEP

The finding of a ‘good’ QoL subgroup, with average QoL scores that mirror scores reported by healthy controls, contradicts the common finding that the QoL of FEP patients is significantly diminished and highlights the importance of approaching QoL in an individualistic manner [31]. The maintenance of good QoL in FEP may be attributable to the protective function of particular personality traits, such as persistent optimism and extraversion despite being faced with adversities [32, 33]. Such traits may make it easier for individuals with FEP to maintain their social support and networks, contributing to more positive evaluations of themselves and their surroundings.

The ‘intermediate’ subgroup mirrors average QoL scores reported by young people in the recovery phase of FEP who do not have a comorbid major depressive disorder, substance disorder, or personality disorder [13]. Interestingly, the strengths (i.e. physical health QoL) and weaknesses (i.e. psychological QoL) of the ‘intermediate’ subgroup at follow-up were the same at baseline [5]. The ‘intermediate’ subgroup was also the largest at baseline and follow-up, suggesting that many young people recovering from FEP may struggle the most with psychological QoL during the early and later stages of recovery.

The ‘poor’ subgroup at follow-up demonstrates that low QoL is an enduring issue for a subset of young people recovering from FEP, despite months of treatment and time for symptomatic recovery. The participants in the ‘poor’ subgroup exhibited similar QoL scores reported by recovering FEP participants with comorbid major depressive disorder in previous studies [13]. This similarity is unsurprising as the ‘poor’ subgroup presented with the highest depressive symptoms and lowest social inclusion at follow-up.

## Factors associated with QoL in FEP

Consistent with previous research on QoL in FEP recovery [34], negative symptoms were negatively correlated with QoL at follow-up. Interestingly, Liao et al. [5] did not find an association between negative symptoms and the subgroups at baseline. Symptom persistence, rather than severity, has been found to contribute to diminished QoL in FEP, which may explain the emergence of a relationship between negative symptoms and QoL subgroups at a later stage of recovery [8]. It is also important to note the phenomenological overlap between negative symptoms and depressive symptoms [35, 36]. This may contribute to an inflated relationship between negative symptoms and QoL at follow-up, as negative symptoms (e.g. memory and attention problems) may have been captured in the measurement of depressive symptoms. This may also explain why negative symptoms did not significantly predict the QoL subgroups after the psychological items of the WHOQoL-BREF were removed, as these items may also capture negative symptomology.

Although depressive symptoms emerged as the strongest predictor of the QoL subgroups, the relationship between depressive symptoms and QoL subgroups may be inflated due to measurement overlap [37, 38]. As Liao et al. [5] failed to acknowledge, the WHOQoL-BREF and CES-D include items that share overlapping themes. Rocha et al. [37] found that 11 WHOQoL-BREF items exhibited differential item functioning for depression, meaning that individuals who score 16 or higher on the CES-D respond differently on those items, even if they have comparable QoL as someone with a lower CES-D score. As the ‘poor’ subgroup has a mean CES-D score of 25.82 at follow-up, the relationship between depressive symptomology and this QoL subgroup may be exaggerated. Although this may not be the case for the ‘good’ and ‘intermediate’ subgroups, the role of depression in predicting heterogeneity in QoL needs to be interpreted with caution to avoid tautological inferences [38]. Nevertheless, when the psychological domain was removed from the clustering algorithm, depression was still the strongest predictor of the QoL subgroups, suggesting that the relationship may not be purely related to measurement overlap. For instance, depression is highly prominent in FEP both as a superimposed comorbidity and an inextricable symptom domain [36], which may also contribute to the relationship between depression and QoL subgroups.

No cognitive measures were associated with the QoL subgroups at follow-up (before and after the psychological domain was removed), suggesting that cognitive functioning at baseline does not explain variation in QoL at a later stage of the recovery phase. Whilst speculative, early intervention may play a role in subduing the predictive ability of cognitive functioning at baseline. Amoretti et al. [39] found that earlier age of FEP onset predicted greater cognitive impairment, highlighting the importance of early intervention in preventing the long-term impact of cognitive dysfunction. Nevertheless, previous research has shown inconsistent findings relating to cognition and QoL in FEP, thus warranting more targeted investigation into the relationship between cognition and QoL in FEP.

In support of previous findings, social inclusion at follow-up predicted subgroup membership before and after the psychological items were removed [5, 13]. Despite being understudied, social inclusion was one of the strongest predictors of the subgroups, highlighting the importance of social participation and social acceptance in influencing perceptions of QoL [40]. As young people highly value a sense of belonging [41], the protective function of social inclusion could be particularly salient in FEP due to its early age of onset. Interestingly, social and occupational functioning did not predict membership to the clusters after the psychological domain was removed, suggesting that facets of functioning may have initially been measured in the psychological items of the WHOQoL-BREF. Corresponding with Liao et al. [5], employment status was not associated with cluster membership at follow-up, despite previous studies reporting significant relationships [42]. Differences between studies may arise due to varying overall employment rates between studies and cultural differences between study cohorts.

## Strengths and limitations

The current study highlights the use of cluster analysis as an innovative approach to capturing heterogeneity in QoL in an FEP cohort. However, this study is limited by the aims and measures used in the original study, which did not specifically focus on QoL and its correlates [10, 11]. It is possible that other important factors, such as physical comorbidities and the use of psychopharmaceuticals, are also associated with QoL variation in FEP and should be considered in the future studies [43]. As this study investigated post-treatment QoL, it is also possible that variation in treatment responses contributed to QoL heterogeneity. Response trajectories in FEP are highly heterogeneous, with better responses associated with higher cognitive functioning and premorbid functioning [44]. The relationship between treatment response and post-treatment QoL should be explored in the future studies.

As a follow-up analysis, our study also allows comparisons with Liao et al.’s [5] baseline clusters. However, statistically modelling participants’ movements between subgroups over time was outside the scope of the study, as the sample size limited our ability to fit these complex models. Consequently, we cannot conclude what factors contribute to longitudinal changes in QoL. As only one study has attempted to do this [7], more longitudinal studies are required to clarify what factors predict QoL *trajectories* in FEP. Finally, the authors also recognise the methodological limitations of univariate screening for predictive relationships [45, 46]. Although we used a more exploratory alpha level of *p* = .10 to reduce the likelihood of wrongly excluding important variables, future studies should employ other statistical methods such as stepwise regression [46].

## Implications and future directions

We have demonstrated that heterogeneity of QoL in FEP exists at a later stage of FEP recovery. The results highlight that although individuals with FEP may exhibit good QoL, many report lower levels across all four QoL domains, despite months of treatment-as-usual. Currently, the central goal of FEP treatment is to encourage engagement and commence pharmacological and psychological interventions that target symptomatic recovery [47]. However, symptomatic remission does not infer good QoL, as low social inclusion and depression can persist in the absence of psychotic symptoms [13, 36]. As improvements in QoL are often considered more important than symptom resolution by young people with FEP and their families, there is a need for interventions that directly address factors associated with poor QoL. However, more research is required to inform better clinical practice [36, 41]. Our study adds to the literature by identifying heterogeneity in QoL at a later stage of FEP recovery and highlighting the understudied role of social inclusion and depression in predicting QoL in FEP post-treatment. As this study did not statistically map participant movement between QoL subgroups, future research should investigate QoL trajectories in FEP and identify the factors that contribute to deteriorating or improving QoL.

## Supplementary Information

Below is the link to the electronic supplementary material.

Supplementary file1 (DOCX 183 KB)

## Data Availability

Data might be available on request and after appropriate institutional agreements and ethics approvals.
