# Supplementary material for: Heterogeneity of quality of life in the later stages of first-episode psychosis recovery
Source: Qual Life Res. 2022 Nov 15;32(3):769–80. doi: 10.1007/s11136-022-03277-x (PMC9992035; doi:10.1007/s11136-022-03277-x)
Supplement: Supplementary file 1 — Supplementary file1 (DOCX 183 KB) [file 11136_2022_3277_MOESM1_ESM.docx]

**Supplementary Material**

**Heterogeneity of Quality of Life in the Later Stages of First-episode Psychosis Recovery**

Clarke E.L.^a^, Allott K.^b,c^, Anderson J.F.I^a^, Gao C.X.^b,c,d^, Filia, K.M. ^b,c^, Killackey E.^b,c,^ Cotton S.M.^b,c^

1. Melbourne School of Psychological Sciences, The University of Melbourne, VIC 3010, Australia
2. Orygen, Parkville, Melbourne VIC, Australia
3. Centre for Youth Mental Health, University of Melbourne, Parkville, Melbourne VIC, Australia
4. School of Public Health & Preventive Medicine, Monash University, Melbourne, Australia

**Appendix 1**

Pairwise correlation and item-total correlation between WHOQoL-BREF individual item data and total depression score (CESD)

| Domain | Facet | Q1 | Q2 | Q3 | Q4 | Q10 | Q15 | Q16 | Q17 | Q18 | Q5 | Q6 | Q7 | Q11 | Q19 | Q26 | Q20 | Q21 | Q22 | Q8 | Q9 | Q12 | Q13 | Q14 | Q23 | Q24 | Q25 | CESD |
| --- | --- | --- | --- | --- | --- | --- | --- | --- | --- | --- | --- | --- | --- | --- | --- | --- | --- | --- | --- | --- | --- | --- | --- | --- | --- | --- | --- | --- |
| Global items | Overall QoL (Q1) | 1.00 |  |  |  |  |  |  |  |  |  |  |  |  |  |  |  |  |  |  |  |  |  |  |  |  |  | .50 |
| Physical Health | General Health (Q2) | .65 | 1.00 |  |  |  |  |  |  |  |  |  |  |  |  |  |  |  |  |  |  |  |  |  |  |  |  | .49 |
|  | Pain (Q3) | -.22 | -.02 | 1.00 |  |  |  |  |  |  |  |  |  |  |  |  |  |  |  |  |  |  |  |  |  |  |  | -.08 |
|  | Dependence on medicinal substances/medical aids (Q4) | -.12 | .05 | .02 | 1.00 |  |  |  |  |  |  |  |  |  |  |  |  |  |  |  |  |  |  |  |  |  |  | -.24 |
|  | Energy (Q10) | .50 | .42 | -.04 | -.35 | 1.00 |  |  |  |  |  |  |  |  |  |  |  |  |  |  |  |  |  |  |  |  |  | .67 |
|  | Mobility (Q15) | .38 | .48 | -.31 | -.18 | .60 | 1.00 |  |  |  |  |  |  |  |  |  |  |  |  |  |  |  |  |  |  |  |  | .39 |
|  | Sleep (Q16) | .46 | .39 | .01 | .22 | .40 | .09 | 1.00 |  |  |  |  |  |  |  |  |  |  |  |  |  |  |  |  |  |  |  | .35 |
|  | Activities of daily living (Q17) | .58 | .66 | -.20 | -.15 | .71 | .66 | .51 | 1.00 |  |  |  |  |  |  |  |  |  |  |  |  |  |  |  |  |  |  | .64 |
|  | Work capacity (Q18) | .39 | .59 | -.21 | -.16 | .36 | .61 | .14 | .58 | 1.00 |  |  |  |  |  |  |  |  |  |  |  |  |  |  |  |  |  | .29 |
| Psychological | Positive feelings (Q5) | .53 | .50 | -.01 | -.08 | .51 | .48 | .31 | .52 | .34 | 1.00 |  |  |  |  |  |  |  |  |  |  |  |  |  |  |  |  | **.64** |
|  | Spirituality (Q6) | .41 | .38 | -.12 | .15 | .37 | .25 | .32 | .43 | .26 | .71 | 1.00 |  |  |  |  |  |  |  |  |  |  |  |  |  |  |  | **.57** |
|  | Thinking, concentration, memory (Q7) | .18 | .32 | .04 | -.23 | .51 | .37 | .29 | .51 | .24 | .35 | .28 | 1.00 |  |  |  |  |  |  |  |  |  |  |  |  |  |  | **.59** |
|  | Body image/appearance (Q11) | .33 | .50 | .02 | -.07 | .57 | .44 | .28 | .56 | ..35 | .58 | .39 | .26 | 1.00 |  |  |  |  |  |  |  |  |  |  |  |  |  | **.52** |
|  | Self-esteem (Q19) | .65 | .64 | .03 | -.19 | .68 | .44 | .39 | .77 | .44 | .70 | .67 | .38 | .63 | 1.00 |  |  |  |  |  |  |  |  |  |  |  |  | **-68** |
|  | Negative feelings (Q26) | -.36 | -.37 | -.10 | .33 | -.59 | -.15 | -.30 | -.51 | -.19 | -.45 | -.44 | -.48 | -.37 | -.59 | 1.00 |  |  |  |  |  |  |  |  |  |  |  | **-.69** |
| Social relationships | Personal relationships (Q20) | .45 | .41 | .03 | -.28 | .60 | .40 | .30 | .51 | .25 | .62 | .35 | .31 | .40 | .56 | -.43 | 1.00 |  |  |  |  |  |  |  |  |  |  | **.**57 |
|  | Sexual activity (Q21) | .39 | .34 | -.17 | -.06 | .42 | .32 | .20 | .32 | .12 | .51 | .28 | .26 | .37 | .31 | -.31 | .64 | 1.00 |  |  |  |  |  |  |  |  |  | .38 |
|  | Social support (Q22) | .48 | .40 | -.05 | -.06 | .50 | .22 | .41 | .45 | .22 | .49 | .44 | .21 | .29 | .45 | -.40 | .62 | .51 | 1.00 |  |  |  |  |  |  |  |  | .48 |
| Environmental | Freedom, safety and security (Q8) | .35 | .34 | -.02 | -.30 | .51 | .37 | .03 | .37 | .25 | .66 | .45 | .49 | .35 | .50 | -.41 | .56 | .40 | .48 | 1.00 |  |  |  |  |  |  |  | .57 |
|  | Physical environment (Q9) | .32 | .37 | -.06 | -.09 | .46 | .41 | .25 | .35 | .36 | .40 | .22 | .41 | .20 | .30 | -.18 | .41 | .27 | .37 | .52 | 1.00 |  |  |  |  |  |  | .25 |
|  | Financial resources (Q12) | .40 | .45 | .02 | -.01 | .37 | .33 | .25 | 50 | .23 | .38 | .24 | .02 | .45 | .43 | -.31 | .50 | .41 | .38 | .26 | .34 | 1.00 |  |  |  |  |  | .32 |
|  | Opportunities for acquiring new skills (Q13) | .55 | .49 | -.22 | .05 | .33 | .46 | .25 | .38 | .41 | .45 | .36 | .12 | .41 | .41 | -.18 | .34 | .35 | .30 | .38 | .19 | .37 | 1.00 |  |  |  |  | .25 |
|  | Participation/opportunities for leisure (Q14) | .39 | .35 | -.35 | .07 | .38 | .47 | .19 | .42 | .24 | .41 | .20 | .32 | .36 | .29 | -.12 | .44 | .52 | .25 | .34 | .40 | .48 | .49 | 1.00 |  |  |  | .30 |
|  | Home environment (Q23) | .40 | .19 | .19 | .08 | .45 | .11 | .46 | .40 | .07 | .41 | .38 | .11 | .31 | .45 | -.17 | .52 | .23 | .47 | .28 | .38 | .22 | .44 | .37 | 1.00 |  |  | .30 |
|  | Health and social care (Q24) | .28 | .41 | .41 | .16 | .31 | .37 | .28 | .32 | .29 | .42 | .29 | .10 | .19 | .22 | -.04 | .43 | .29 | .56 | .37 | .50 | .38 | .59 | .44 | .66 | 1.00 |  | .23 |
|  | Transport (Q25) | .14 | .22 | .04 | .22 | .25 | .28 | .11 | .34 | .03 | .39 | .17 | .01 | .27 | .22 | .02 | .38 | .26 | .37 | .22 | .28 | .40 | .24 | .29 | .43 | .52 | 1.00 | .15 |

**Appendix 2**

*Name, description and version of R packages*

| R Package | Description | Version |
| --- | --- | --- |
| base | The R base package | 4.1.0 |
| biotools | Tools for biometry and applied statistics in agricultural sciences | 4.2 |
| cluster | “Finding Groups in Data”: Cluster Analysis Extended Rousseeuw et al. | 2.1.2 |
| cluster.datasets | Cluster analysis data sets | 1.0-1 |
| clustree | Visualise clustering at different resolutions | 0.4.3 |
| clValid | Validation of clustering results | 0.7 |
| corrplot | Visualisation of correlation matrix | 0.90 |
| cowplot | streamlined plot theme and plot annotations for ggplot2 | 1.1.1 |
| data.table | Extension of ‘data.frame’ | 1.14.0 |
| datasets | The R datasets package | 4.1.0 |
| dendextend | Extending ‘dendrogram’ functionality in R | 1.15.1 |
| dplyr | A grammar of data manipulation | 1.0.7 |
| factoextra | Extract and visualise the results of multivariate data analysis | 1.0.7 |
| FactoMineR | Multivariate exploratory data analysis and data mining | 2.4 |
| forcats | Tools for working with categorical variables | 0.5.1 |
| foreign | Reading datasets | 0.8-81 |
| GGally | Extension to ‘ggplot2’ | 2.1.2 |
| ggfortify | Data visualisation for tools for statistical analysis | 0.4.12 |
| ggplot2 | Create elegant data visualisations using the gramma of graphics | 3.3.5 |
| ggpubr | ‘ggplot2’ based publication ready plots | 0.4.0 |
| ggraph | An implementation of grammar of graphics for graphs and networks | 2.0.5 |
| graphics | The R graphics package | 4.1.0 |
| haven | Import and export ‘SPSS’; ‘Stata’ and ‘SAS’ files | 2.4.1 |
| kableExtra | Construct complex table with ‘kable’ and pipe syntax | 1.3.4 |

| R Package | Description | Version |
| --- | --- | --- |
| knitr | A general-purpose package for dynamic report generation in R | 1.33 |
| lsr | Companion to ‘Learning Statistics with R’ | 0.5 |
| magrittr | A forward-pipe operated in R | 2.0.1 |
| NbClust | Determining the best number of clusters in a data set | 3.0 |
| nnet | Feed-forward neural networks and multinominal log-linear models | 7.3-16 |
| philentropy | Similarity and distance quantification between probability functions | 0.5.0 |
| profileR | Profile analysis of multivariate data in R | 0..3-5 |
| psych | Procedures for psychological, psychometric, and personality research | 2.1.6 |
| stats | The R stats package | 4.1.0 |
| questionr | Functions to make survey processing easier | 0.7.4 |
| readr | Read rectangular text data | 2.0.0 |
| reshape2 | Flexibility reshape data: A reboot of the reshape package | 1.4.4 |
| tibble | Simple data frames | 3.1.3 |
| tidyr | Tidy messy data | 1.1.3 |
| tidyverse | Easily install and load the ‘Tidyverse’ | 1.3.1 |

*Notes*. Descriptions are taken straight from RStudio (Version 1.4.1717).

**Appendix 3**

*Comparison of the demographic, clinical characteristics, cognition and QoL of participants by follow-up availability*

| Measures | Follow-up  (*N* =102) | No follow-up  (*N* =43) | *t* | $x^{2}$ | *df* | *p-*value | *Effect-size* |
| --- | --- | --- | --- | --- | --- | --- | --- |
| Demographics |  |  |  |  |  |  |  |
| *Age*, mean (*SD*) | 20.25 (2.38) | 20.72 (2.37) | -.91 | - | 77.1 | . 367 | .020 |
| *Gender*, male (%) | 66 (64.71) | 36 (83.72) | - | 3.24 | 1 | .072 | .149 |
| Currently employed (%) | 19 (18.63) | 5 (11.33) | - | .63 | 1 | .429 | .066 |
| Clinical characteristics |  |  |  |  |  |  |  |
| *DUP*, mean (*SD*) | 245.18 (421.33) | 353.75 (616.98) | -1.01 | - | 55.4 | .315 | .206 |
| *Treatment group,* vocational (%) | 56 (54.90) | 16 (37.21) | - | 3.11 | 1 | .077 | .146 |
| Positive symptoms, mean (*SD*) | 8.17(4.14) | 9.16 (4.65) | -1.22 | - | 71.3 | .228 | .223 |
| Negative symptoms, mean (*SD*) | 25.18 (12.67) | 25.70 (11.51) | -.24 | - | 86.5 | .811 | . 043 |
| Depressive symptoms, mean (*SD*) | 19.57 (11.50) | 20.86 (12.25) | -.59 | - | 74.7 | .556 | .109 |
| Cognition |  |  |  |  |  |  |  |
| IQ, mean (*SD*) | 93.17 (15.12) | 91.93 (14.82) | .47 | - | 80.49 | .750 | .083 |
| ToM, mean (*SD*) | 4.54 (1.29) | 4.27 (1.50) | 1.03 | - | 69.20 | . 308 | .193 |
| Semantic verbal fluency, mean (*SD*) | 18.96 (6.08) | 18.12 (5.22) | .85 | - | 91.29 | .400 | .148 |
| QoL |  |  |  |  |  |  |  |
| Physical health, mean (*SD*) | 65.79 (16.30) | 62.04 (15.59) | 1.30 | - | 82.4 | .196 | .235 |
| Psychological, mean (*SD*) | 53.59 (19.76) | 52.81 (19.32) | .22 |  | 80.7 | .825 | .040 |
| Social relationships, mean (*SD*) | 57.76 (21.34) | 56.40 (23.56) | .33 | - | 72.5 | .744 | .061 |
| Environmental, mean (*SD*) | 63.60 (14.81) | 58.07 (17.98) | 1.78 | - | 67.2 | .080 | .334 |

*Notes*. *DUP* = duration of untreated psychosis; ToM = Theory of Mind. Measures that are in italics were taken at baseline. Effect sizes were calculated using Cohen’s d and Phi coefficient.

**Appendix 4**

*Follow-up hierarchical agglomerative clustering dendrograms for various cluster solutions*


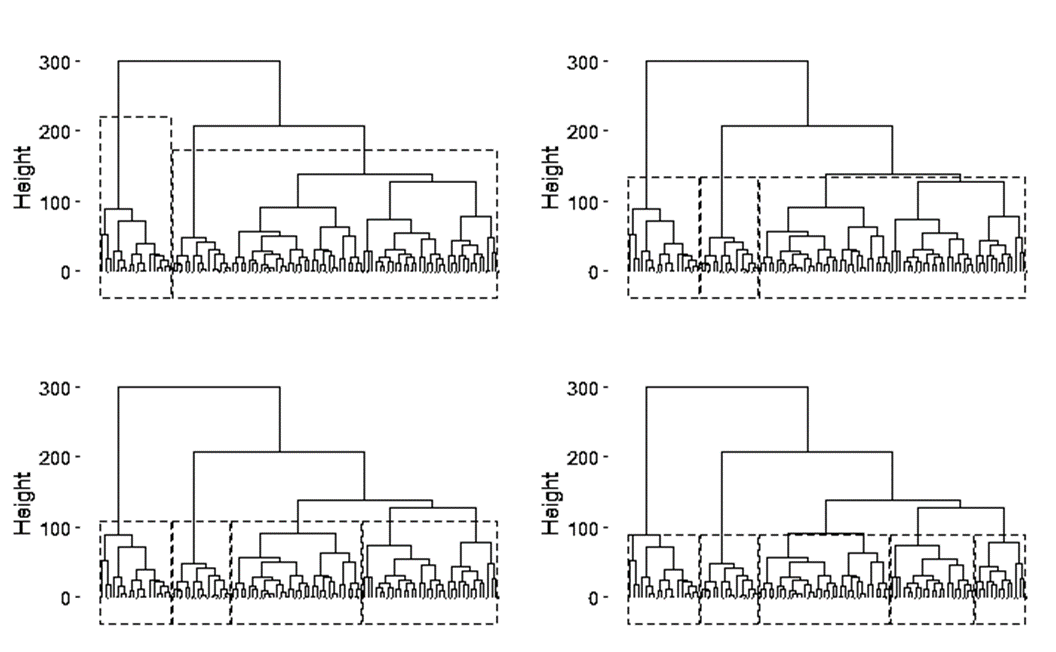


*Notes*. The height on the y-axes displays the distances between participants’ QoL observations and the horizontal bars indicate the point at which participants were merged into a cluster based on such distances. As the horizontal bars become wider, the distances between the grouped participants enlarges. The dashed line represents where the dendrogram would be ‘cut’ based on the relevant cluster solution.

**Appendix 5**

*Follow-up hierarchical agglomerative clustering scree plot*


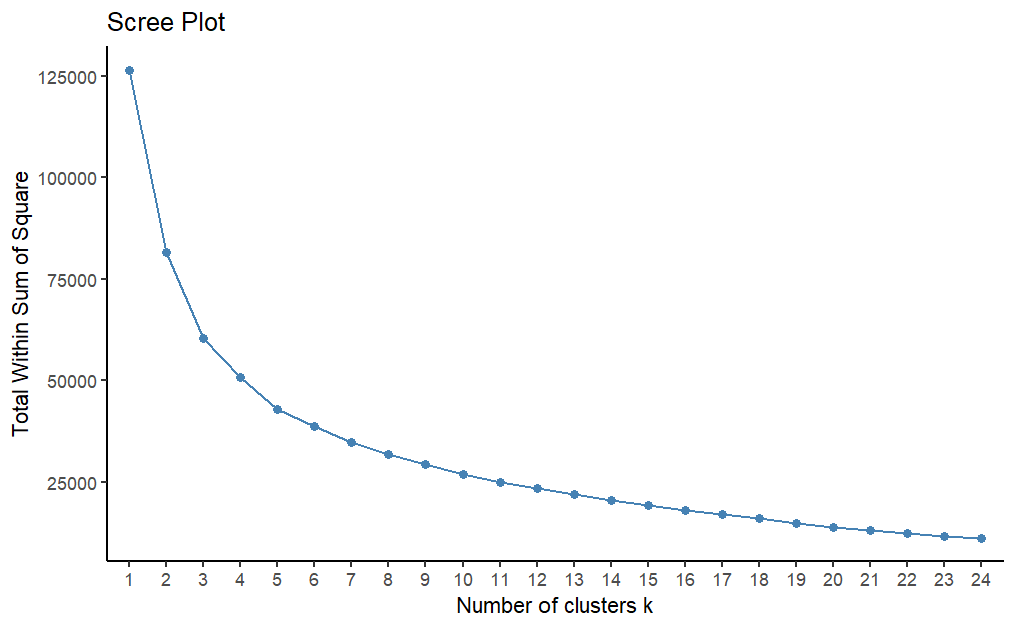


**Appendix 6**

*One-way ANOVA results using each QoL domain as the criterion*

| QoL domain | Predictor | Sum of Squares | *df* | Mean Square | *F* | *p-*value | η^2^ |
| --- | --- | --- | --- | --- | --- | --- | --- |
| Physical health |  |  |  |  |  |  |  |
|  | Cluster group | 7463 | 2 | 3731 | 33.4 | <.001* | .41 |
|  | Error | 10851 | 97 | 112 |  |  |  |
| Psychological |  |  |  |  |  |  |  |
|  | Cluster group | 17216 | 2 | 8608 | 140.7 | <.001* | 56 |
|  | Error | 13647 | 97 |  |  |  |  |
| Social relationships |  |  |  |  |  |  |  |
|  | Cluster group | 34929 | 2 | 17465 | 100.3 | <.001* | .67 |
|  | Error | 16893 | 97 | 174 |  |  |  |
| Environmental |  |  |  |  |  |  |  |
|  | Cluster group | 12604 | 2 | 6302 | 47.3 | <.001* | .49 |
|  | Error | 12914 | 97 | 133 |  |  |  |

Notes*.* ** p* = < .001

**Appendix 7**

*Comparison of baseline and follow-up cluster groups on each QoL domain*

| Cluster group | Baseline | Follow-up | *t* | *df* | *p*-value | Cohen*’s d* |
| --- | --- | --- | --- | --- | --- | --- |
|  | Mean (*SD*) | Mean (*SD*) |  |  |  |  |
| ‘Good’ cluster |  |  |  |  |  |  |
| Physical health | 79.25 (10.85) | 81.68 (9.92) | -.99 | 67.73 | .324 | .23 |
| Psychological | 75.20 (9.87) | 78.90 (10.20) | -1.55 | 63.55 | .125 | .37 |
| Social relationships* | 77.38 (13.31) | 85.75 (11.63) | -2.86 | 68.92 | .006* | .66 |
| Environmental | 77.23 (11.01) | 80.75 (10.05) | -1.42 | 67.77 | .161 | .33 |
| ‘Intermediate’ cluster |  |  |  |  |  |  |
| Physical health | 65.65 (10.39) | 68.38 (10.31) | -1.38 | 101.7 | .171 | .26 |
| Psychological* | 51.52 (11.26) | 58.16 (13.20) | -2.79 | 92.07 | .006* | .55 |
| Social relationships* | 58.99 (13.58) | 64.93 (13.20) | -2.32 | 102.72 | .022* | . 44 |
| Environmental* | 60.91 (11.00) | 66.27 (11.75) | -2.45 | 97.71 | .016* | .47 |
| Poor cluster |  |  |  |  |  |  |
| Physical health* | 47.86 (12.04) | 57.82 (12.04) | -3.07 | 40.77 | .004* | .83 |
| Psychological* | 33.33 (13.41) | 57.83 (10.80) | -3.00 | 49.03 | .004* | .76 |
| Social relationships | 33.75 (17.08) | 32.94 (15.25) | .19 | 45.02 | .850 | .05 |
| Environmental | 47.58 (12.45) | 49.11 (13.04) | -0.44 | 39.13 | .661 | .12 |

*Notes*. * = *p* < .05

**Appendix 8**

*Bivariate associations between external baseline variables and follow-up cluster group membership*

|  | Contrast – ‘poor’ vs ‘good | | | | | Contrast – ‘intermediate’ vs ‘good’ | | | | |
| --- | --- | --- | --- | --- | --- | --- | --- | --- | --- | --- |
|  |  | 95% CI of OR | |  |  |  | 95% CI of OR | |  |  |
| Baseline Measures | OR | LCI | UCI | Wald | *p* | OR | LCI | UCI | Wald | *p* |
| Clinical Characteristics |  |  |  |  |  |  |  |  |  |  |
| *DUP* | 1.00 | 1.00 | 1.00 | 1.86 | .063 | 1.00 | 1.00 | 1.00 | 1.40 | .163 |
| Positive symptoms (BPRS) | 1.27 | 1.09 | .148 | 3.06 | .002 | 1.12 | .98 | .1.27 | 1.67 | .094 |
| Negative symptoms (SANS) | 1.02 | .98 | 1.07 | .92 | .354 | .99 | .95 | 1.03 | -.52 | .600 |
| Depressive symptoms (CESD) | 1.08 | 1.02 | 1.14 | .03 | .004 | 1.03 | 0.98 | 1.07 | 1.12 | .262 |
| Vocational Intervention (Yes) | 1.73 | .56 | 5.35 | 0.96 | .339 | 1.26 | .510 | 3.12 | .50 | .615 |
| Cognition |  |  |  |  |  |  |  |  |  |  |
| IQ | .99 | .95 | 1.03 | -.60 | .546 | 1.00 | .97 | 1.03 | -.25 | .804 |
| ToM | .92 | .60 | 1.42 | -.37 | .720 | .96 | .67 | 1.37 | -.22 | .824 |
| Semantic verbal fluency | .98 | .89 | 1.07 | -.43 | .670 | 1.00 | .93 | 1.08 | -.00 | .999 |

*Notes.* BPRS = Brief Psychiatric Rating Scale – Positive Symptoms Subscale; CESD = Centre for Epidemiological Studies – Depression scale; CI = confidence interval; DUP = duration of untreated psychosis; LCI = lower confidence interval; OR = odds ratio; SANS = Scale for the Assessment of Negative Symptoms; ToM = Theory of Mind; UCI = upper confidence interval. Measures that are in italics are premorbid variables, taken at baseline.

* = *p* < .100

**Appendix 9**

*Bivariate associations between external variables at follow-up and follow-up cluster group membership*

|  | Contrast – ‘poor’ vs ‘good | | | | | Contrast – ‘intermediate’ vs ‘good’ | | | | |
| --- | --- | --- | --- | --- | --- | --- | --- | --- | --- | --- |
|  |  | 95% CI of OR | |  |  |  | 95% CI of OR | |  |  |
| Follow-up variable | OR | LCI | UCI | Wald | *p*-value | OR | LCI | UCI | Wald | *p*-value |
| Clinical characteristics |  |  |  |  |  |  |  |  |  |  |
| Positive symptoms (BPRS)* | 1.24 | 1.07 | 1.43 | 2.85 | .004* | 1.06 | .92 | 1.22 | .78 | .438 |
| Negative symptoms (SANS)* | 1.08 | 1.03 | 1.12 | 3.56 | < .001* | 1.03 | 1.00 | 1.07 | 1.94 | .052* |
| Depressive symptoms (CESD)* | 1.40 | 1.24 | 1.59 | 5.27 | < .001* | 1.24 | 1.11 | 1.38 | 3.94 | <.001* |
| Functioning |  |  |  |  |  |  |  |  |  |  |
| Employment status (No) | 1.81 | .55 | 5.91 | .98 | .329 | 1.20 | .48 | 3.03 | .47 | .694 |
| Social/occupational functioning (SOFAS)* | .94 | .90 | .99 | -2.62 | .009* | .98 | .94 | 1.00 | -1.46 | .145 |
| Social inclusion* | .72 | .63 | 0.82 | -4.93 | < .001* | 0.82 | .74 | .91 | -3.74 | < .001* |

*Notes**.* BPRS = Brief Psychiatric Rating Scale – Positive Symptoms Subscale; CESD = Centre for Epidemiological Studies –Depression scale; CI = confidence interval; LCI = lower confidence interval; OR = odds ratio; SANS = Scale for the Assessment of Negative Symptoms; SOFAS = Social and Occupational Functioning Assessment Scale; UCI = upper confidence interval

* = *p* < .100


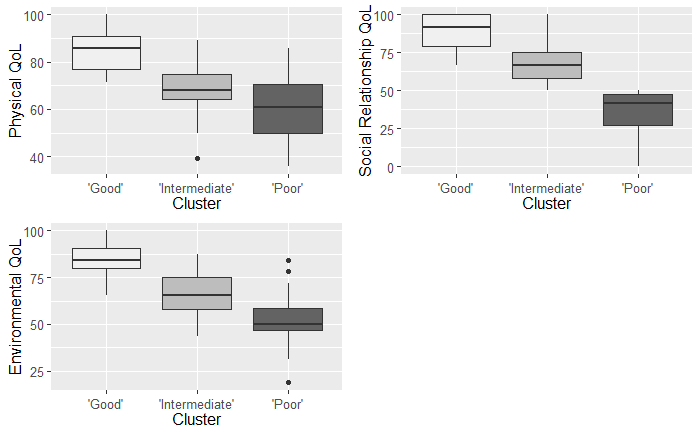


**Appendix 10**

*QoL profiles for each cluster group at 18-months follow-up after the Psychological items were removed (sensitivity analysis)*

Notes. The ‘good’ group (23%; *n* = 23), ‘intermediate’ (51%; *n* = 51) and ‘poor’ (26%; *n* = 26) exhibited different QoL profile

**Appendix 11**

*One-way ANOVA results using each QoL domain as the criterion in cluster group membership with Psychological domain excluded (sensitivity analysis)*

| Physical health |  |  |  |  |  |  |  |
| --- | --- | --- | --- | --- | --- | --- | --- |
|  | Cluster group | 7472 | 2 | 3736 | 33.4 | <.001* | .41 |
|  | Error | 10841 | 97 | 117 |  |  |  |
| Social relationships |  |  |  |  |  |  |  |
|  | Cluster group | 37280 | 2 | 18640 | 124.3 | <.001* | .72 |
|  | Error | 14543 | 97 | 150 |  |  |  |
| Environmental |  |  |  |  |  |  |  |
|  | Cluster group | 12282 | 2 | 6141 | 45.0 | <.001* | .48 |
|  | Error | 13236 | 97 | 136 |  |  |  |

Notes*.* ** p* = < .001

**Appendix 12**

*Univariate associations between external variables and cluster group membership with Psychological domain excluded (sensitivity analysis)*

|  | Contrast – ‘poor’ vs ‘good | | | | | Contrast – ‘intermediate’ vs ‘good’ | | | | |
| --- | --- | --- | --- | --- | --- | --- | --- | --- | --- | --- |
|  |  | 95% CI of OR | |  |  |  | 95% CI of OR | |  |  |
| Variable | OR | LCI | UCI | Wald | *p*-value | OR | LCI | UCI | Wald | *p*-value |
| Clinical characteristics |  |  |  |  |  |  |  |  |  |  |
| *DUP* | 1.00 | 1.00 | 1.00 | 1.39 | .166 | 1.00 | 1.00 | 1.00 | 1.11 | .264 |
| *Treatment group* | 1.73 | .54 | 5.59 | .92 | .359 | .63 | .23 | 1.70 | -.91 | .364 |
| *Baseline positive symptoms* (BPRS) | 1.31 | 1.09 | 1.57 | 2.94 | .003* | 1.20 | 1.01 | 1.42 | 2.11 | 0.03* |
| Positive symptoms (BPRS)* | 1.34 | 1.07 | 1.68 | 1.56 | .010* | 1.19 | .96 | 1.48 | 2.58 | . 119 |
| *Baseline negative symptoms* (SANS) | 1.03 | .99 | 1.08 | 1.33 | .185 | 1.01 | .97 | 1.05 | 0.35 | .723 |
| Negative symptoms (SANS)* | 1.08 | 1.03 | 1.13 | 3.41 | .001* | 1.04 | 1.00 | 1.08 | 2.13 | .033* |
| *Baseline depressive symptoms* (CESD)* | 1.07 | 1.02 | 1.13 | 2.56 | .010* | 1.07 | .97 | 1.07 | 0.65 | .519 |
| Follow-up depressive symptoms (CESD)* | 1.43 | 1.23 | 1.65 | 4.75 | <.001* | 1.30 | 1.13 | 1.49 | 3.76 | <.001* |
| Functioning |  |  |  |  |  |  |  |  |  |  |
| Social/occupational functioning (SOFAS)* | .93 | .88 | .97 | -3.26 | .001* | .96 | .93 | 1.00 | -2.07 | .039* |
| Social inclusion* | .74 | .65 | .84 | -4.47 | <.001* | .81 | .72 | .91 | -3.52 | <.001* |
| Employment | 2.09 | .63 | 6.90 | 1.21 | .228 | 1.19 | .44 | 3.23 | .34 | .730 |
| Cognition |  |  |  |  |  |  |  |  |  |  |
| *IQ, mean (SD)* | .99 | .96 | 1.03 | -0.42 | .674 | .97 | .94 | 1.01 | -1.59 | .111 |
| *ToM, mean (SD)* | .86 | .54 | 1.37 | -0.63 | .526 | .75 | .49 | 1.13 | -1.38 | .168 |
| *Semantic verbal fluency, mean (SD)* | .98 | . 89 | 1.07 | -0.43 | .664 | .96 | .88 | 1.04 | -1.03 | .303 |

*Notes.* BPRS = Brief Psychiatric Rating Scale – Positive Symptoms Subscale; CESD = Centre for Epidemiological Studies –Depression scale; CI = confidence interval; LCI = lower confidence interval; OR = odds ratio; SANS = Scale for the Assessment of Negative Symptoms; SOFAS = Social and Occupational Functioning Assessment Scale; UCI = upper confidence interval. Measures that are in italics were taken at baseline.

* = *p* < .01

**Appendix 13**

*Multivariate associations between external variables and cluster group membership with Psychological domain excluded (sensitivity analysis)*

|  | Contrast – ‘poor’ vs ‘good | | | | | Contrast – ‘intermediate’ vs ‘good’ | | | | |
| --- | --- | --- | --- | --- | --- | --- | --- | --- | --- | --- |
|  |  | 95% CI of OR | |  |  |  | 95% CI of OR | |  |  |
| Variable | OR | LCI | UCI | Wald | *p*-value | OR | LCI | UCI | Wald | *p*-value |
| Clinical characteristics |  |  |  |  |  |  |  |  |  |  |
| *Baseline positive symptoms* (BPRS) | 1.16 | .86 | 1.57 | .95 | .343 | 1.23 | .94 | 1.62 | 1.44 | .149 |
| Positive symptoms (BPRS) | 1.03 | .81 | 1.29 | .22 | .824 | 0.98 | .79 | 1.21 | -.22 | .828 |
| Negative symptoms (SANS)* | 1.06 | .98 | 1.15 | 1.44 | .151 | 1.02 | 1.02 | 1.09 | .71 | .477 |
| *Baseline depressive symptoms* (CESD) | 1.07 | .97 | 1.16 | 1.39 | .163 | 1.00 | .92 | 1.07 | -0.13 | .895 |
| Follow-up depressive symptoms (CESD)* | 1.37 | 1.15 | 1.63 | 3.54 | <.001* | 1.30 | 1.10 | 1.52 | 3.11 | .002* |
| Functioning |  |  |  |  |  |  |  |  |  |  |
| Social/occupational functioning (SOFAS) | 1.01 | .93 | 1.09 | 0.21 | .835 | .99 | .94 | 1.06 | -.19 | .853 |
| Social inclusion* | .77 | .63 | .95 | -2.51 | .012* | .80 | .66 | .96 | -2.40 | .017* |

*Notes.* BPRS = Brief Psychiatric Rating Scale – Positive Symptoms Subscale; CESD = Centre for Epidemiological Studies –Depression scale; CI = confidence interval; LCI = lower confidence interval; OR = odds ratio; SANS = Scale for the Assessment of Negative Symptoms; SOFAS = Social and Occupational Functioning Assessment Scale; UCI = upper confidence interval. Measures that are in italics were taken at baseline.

* = *p* < .05
